# Longitudinal Instrumental Assessment and Management of Dysphagia in a Child with Distal Trisomy 13q: A Case Report

**DOI:** 10.3390/diagnostics16213652

**Published:** 2026-11-05

**Authors:** Abdullah Mohammed Alfaris, Shaden Abdulmohsen Alabdulkarim, Abdulmohsen Abdulkarim Alanazi, Anas Osman Hamdoun, Hanoof Abdullah Alaboodi, Marzouk Sari Alotaibi, Rima Khaled Alzahrani, Hissa Abdullah Alhajri, Shahad M. Tayyash, Nisreen Naser Al Awaji

**Affiliations:** 1 Rehabilitation Department, King Abdullah Bin Abdulaziz University Hospital, Riyadh 11552, Saudi Arabia; amalfaris@kaauh.edu.sa (A.M.A.); saalabdulkarim@kaauh.edu.sa (S.A.A.); abaalanazi@kaauh.edu.sa (A.A.A.); aohamdoun@kaauh.edu.sa (A.O.H.); haalaboodi@kaauh.edu.sa (H.A.A.); msalotaibi@kaauh.edu.sa (M.S.A.); 2 Department of Health Communication Sciences, College of Health and Rehabilitation Sciences, Princess Nourah bint Abdulrahman University, Riyadh 11671, Saudi Arabia; reemakmazz@outlook.com (R.K.A.); s.tayyash@gmail.com (S.M.T.); 3 Fad Center, Anas Ibn Malik Road, Al Narjis, Riyadh 12130, Saudi Arabia; hessaalhajri43@gmail.com

**Keywords:** pediatric dysphagia, modified barium swallow study, videofluoroscopic swallowing study, silent aspiration, distal trisomy 13q, instrumental assessment, gastrostomy

## Abstract

**Background and Clinical Significance:** Children with rare neurogenetic disorders may present with complex feeding and swallowing impairments requiring repeated assessment and individualized management. In severe pediatric dysphagia, instrumental evaluation is particularly important for identifying aspiration, characterizing swallowing abnormalities, and informing decisions regarding oral feeding. **Case Presentation:** We report the longitudinal assessment and management of dysphagia in a 6-year-old girl with genetically confirmed distal trisomy 13q who presented with severe feeding difficulties and prolonged gastrostomy-tube dependence. Serial clinical evaluations and modified barium swallow studies (MBSS), performed between 18 months and six years of age, documented persistent oropharyngeal dysphagia involving both oral and pharyngeal phases. Earlier examinations demonstrated aspiration across tested consistencies, including silent aspiration. Management included structured swallowing therapy, controlled oral trials, texture modification, and continued enteral nutritional support, with feeding recommendations informed by clinical and instrumental reassessments. At 5 years and 10 months, aspiration persisted with thin liquids, although no aspiration was observed with thickened liquids or purees during the assessed trials. At six years of age, no aspiration was observed under the tested conditions, although deep laryngeal penetration and other pharyngeal swallowing abnormalities persisted. These findings supported cautious progression of oral intake while gastrostomy feeding remained necessary. **Conclusions:** This case highlights the value of longitudinal instrumental swallowing assessment in guiding individualized feeding decisions in children with complex neurodevelopmental disorders. Although swallowing findings changed over time, persistent airway protection abnormalities and differences in assessment conditions underscore the need for cautious interpretation and continued multidisciplinary management.

## 1. Introduction

Pediatric dysphagia arises from diverse neurological, structural, and developmental conditions and can adversely affect growth, respiratory health, and quality of life [1,2]. In children with rare chromosomal disorders, dysphagia is often multifactorial, involving hypotonia, craniofacial anomalies, and impaired neuromotor coordination of swallowing [3,4]. Feeding and swallowing difficulties may also involve overlapping medical, nutritional, and feeding-skill concerns, highlighting the importance of multidisciplinary assessment and management [5]. Clinical feeding evaluations provide important information about oral feeding skills and observable signs of swallowing difficulty; however, aspiration, particularly silent aspiration, may not be reliably identified through clinical assessment alone [6,7]. Instrumental swallowing assessment therefore plays an important role in characterizing airway invasion and informing feeding recommendations in children with suspected oropharyngeal dysphagia [8]. Detailed longitudinal descriptions of swallowing impairment based on serial instrumental evaluation remain limited for many rare chromosomal disorders.

Partial trisomy 13q results from copy-number gain involving a segment of the long arm of chromosome 13, with the clinical phenotype varying according to the extent and location of the duplicated region and the presence of additional chromosomal abnormalities. Clinical manifestations may include growth restriction, intellectual disability, global developmental delay, congenital anomalies, and feeding difficulties [9,10]. In the absence of condition-specific evidence, dysphagia management must therefore draw on broader principles of pediatric dysphagia assessment and intervention [4,11].

This report describes the longitudinal swallowing course of a 6-year-old girl with distal trisomy 13q, based on serial clinical and instrumental assessments from infancy through childhood. The case illustrates how repeated assessment and multidisciplinary management informed feeding recommendations and therapeutic progression over time. To our knowledge, detailed longitudinal descriptions of dysphagia management in children with distal trisomy 13q remain scarce.

## 2. Case Presentation

A 6-year-old girl with a confirmed diagnosis of distal trisomy 13q presented with a complex medical history beginning in early infancy. She exhibited global developmental delay from early infancy and developed a seizure disorder at three months of age, initially characterized by hypsarrhythmia and later progressing to multiple seizure types. Severe hypotonia was also noted as part of her clinical presentation. Seizure management required several anti-seizure medications, including phenobarbital, clobazam, and rufinamide. Craniofacial and other congenital anomalies were noted throughout the clinical course. She was also diagnosed with bilateral myopia and hearing impairment, for which she regularly used corrective eyeglasses and hearing aids.

Prenatal history was notable for two umbilical arteries and oligohydramnios. She was born at term with a birth weight of 2.8 kg. There was no parental consanguinity, and both parents were healthy (maternal age: 32 years, paternal age: 40 years). Her younger brother demonstrated typical development. Dysmorphic features included ankyloglossia (tongue-tie) and postaxial polydactyly of the left hand. She was followed by cardiology for a small patent ductus arteriosus (PDA) and by urology for recurrent urinary tract infections. Feeding difficulties were prominent from infancy, with multiple hospital admissions due to aspiration pneumonia. Swallowing dysfunction and silent aspiration, particularly with thin liquids, were documented during her clinical course. She also received treatment for gastroesophageal reflux disease (GERD).

Genetic investigations initially included whole-exome sequencing and an epilepsy gene panel, which were nondiagnostic. Subsequent whole-genome sequencing identified a pathogenic terminal copy-number gain of approximately 39 Mb involving chromosome 13q22.2–q34 (copy-number state 3), consistent with distal trisomy 13q. This finding was confirmed by chromosomal microarray analysis (CMA). CMA is recommended as a first-tier diagnostic test for individuals with developmental disabilities or congenital anomalies [12]. An additional approximately 3 Mb mosaic copy-number gain involving 13q21.32–q21.33 and three smaller copy-number gains involving distal chromosome 12q were identified and classified as variants of uncertain significance. Conventional chromosome analysis subsequently demonstrated a female karyotype of 47, XX, add (12) (q24.31), +mar, indicating additional chromosomal material of undetermined origin on chromosome 12 and a small supernumerary marker chromosome. The precise structural relationship between these cytogenetic findings and the detected copy-number gains could not be established from the available investigations.

The patient was first referred to the swallowing team at 13 months of age due to persistent feeding difficulties. Clinical swallowing assessment, including an oral motor examination (OME) and bedside swallowing evaluation, revealed poor lip seal, tongue thrust, anterior and lateral spillage, weak tongue movements, reduced jaw stability, limited base-of-tongue movement, diminished laryngeal elevation, delayed pharyngeal response, slow swallow transit, poor oral control, and intermittent coughing during thickened-liquid trials. These findings suggested impairment of both the oral and pharyngeal swallowing phases. At that time, she was dependent on a nasogastric tube (NGT) for feeding. Recurrent episodes of cyanosis and stridor during NGT feeds prompted further investigation.

**Figure 1:**
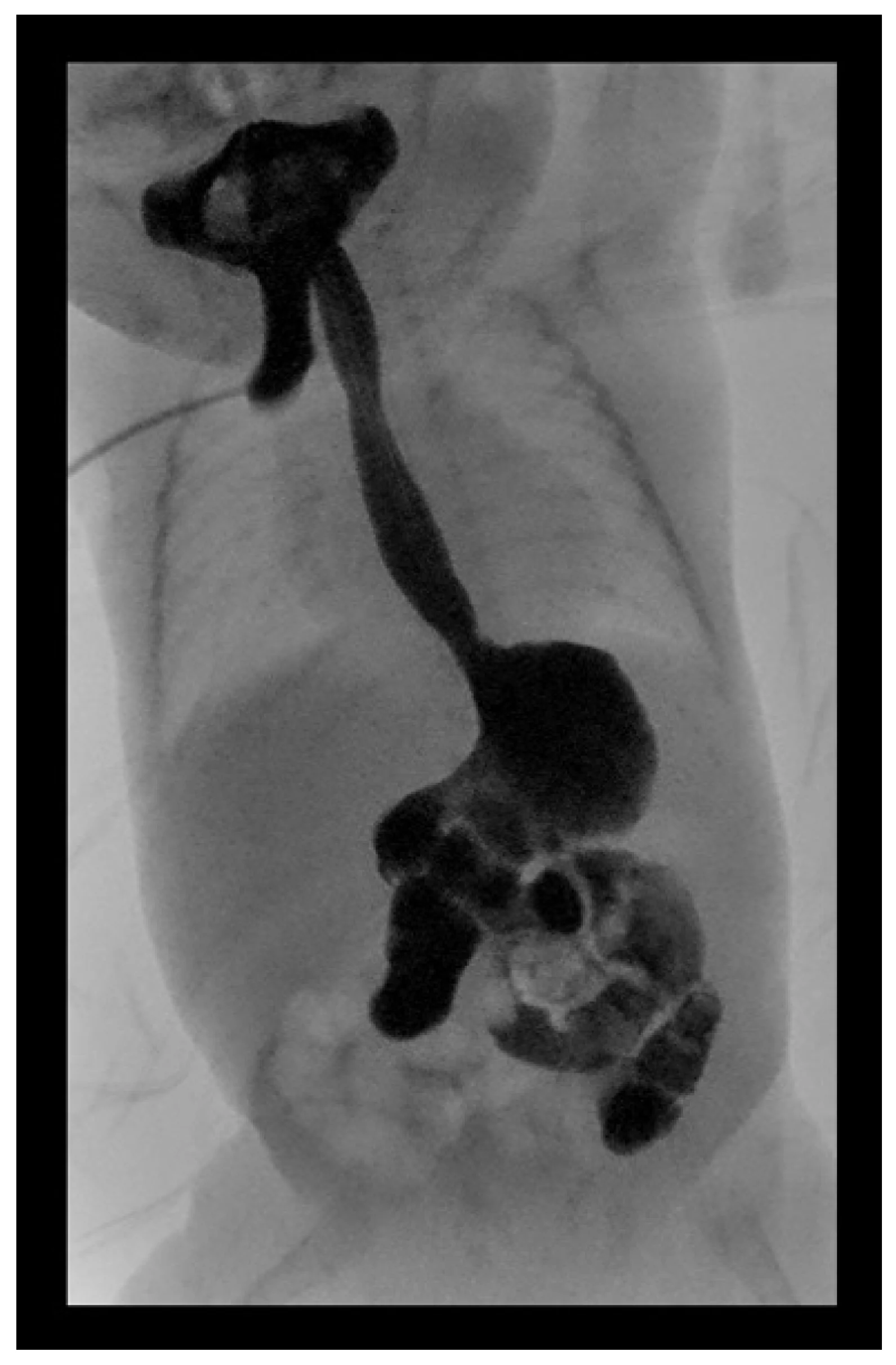
Lateral view from the Barium Swallow Study at 13 months showing unobstructed esophageal passage with severe gastroesophageal reflux extending to the upper esophagus.

**Figure 2:**
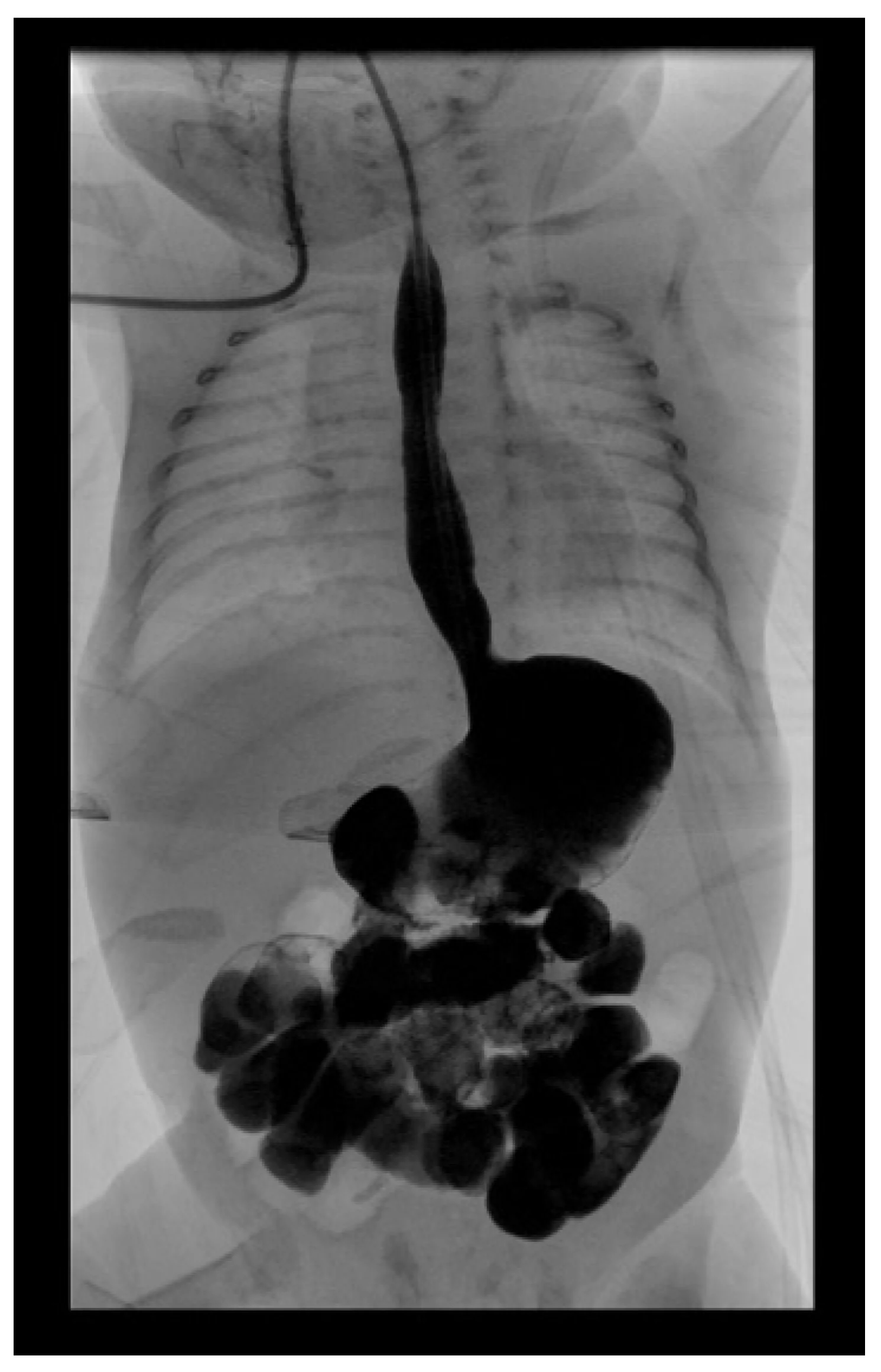
Frontal radiographic view from the Barium Swallow Study performed at 13 months of age, showing normal positioning of the duodenojejunal (DJ) junction.

A Barium Swallow Study performed with diluted water-soluble contrast demonstrated unobstructed passage through the esophagus, stomach, and duodenum, with normal duodenojejunal junction positioning and no structural abnormalities or contrast aspiration observed (Figure 1 and Figure 2). However, severe GERD was observed, with reflux extending to the upper esophagus and oral cavity.

**Figure 3:**
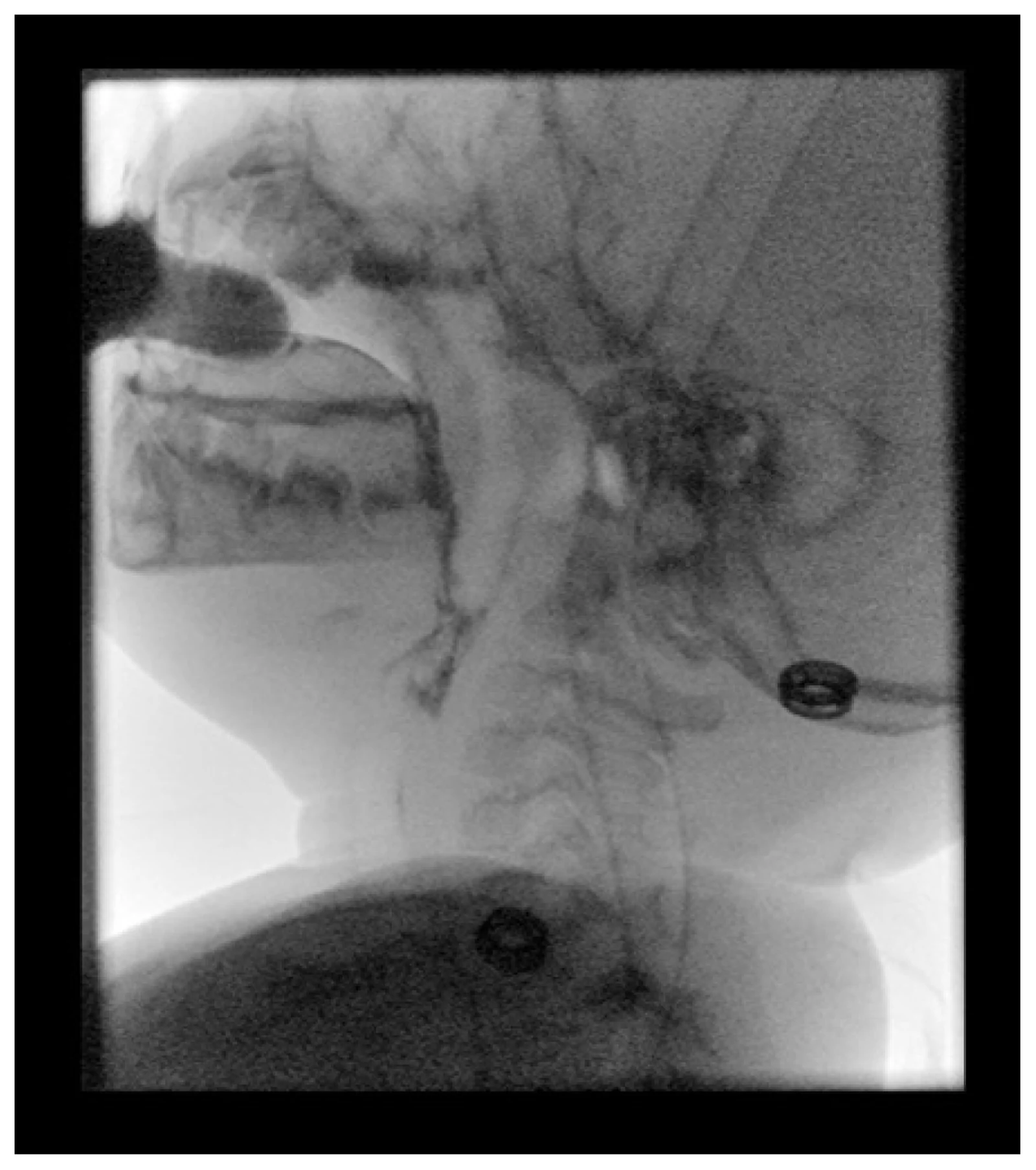
Lateral fluoroscopic image from the MBSS performed at 18 months of age, demonstrating premature bolus spillage and aspiration of thin liquid. The arrow indicates the aspirated contrast material.

Given concerns about swallowing efficiency and safety, an MBSS was performed at 18 months of age. The examination demonstrated severe oropharyngeal dysphagia with aspiration of thin liquids, thickened liquids, and purees (Figure 3). A Penetration–Aspiration Scale (PAS) score of 8 was documented, indicating aspiration without an attempt to eject the material. Thin liquids were administered using a milk bottle. Thickened liquid was prepared using one scoop of thickening agent mixed with 300 mL of milk and was also administered using a milk bottle, while 1 mL of puree was administered by syringe. Additional findings included significant vallecular and pyriform sinus residue, premature bolus spillage, delayed swallow initiation, and coating of the posterior pharyngeal wall. Based on these findings, gastrostomy tube (G-tube) feeding was recommended. Additional examination details are summarized in Table 2.

At 22 months of age, a Barium Meal Study was performed to further evaluate severe GERD and assess the need for fundoplication. Imaging demonstrated normal esophageal morphology, appropriate positioning to the duodenojejunal junction, and adequate bowel opacification, with findings supporting persistent reflux despite medical therapy.

**Figure 4:**
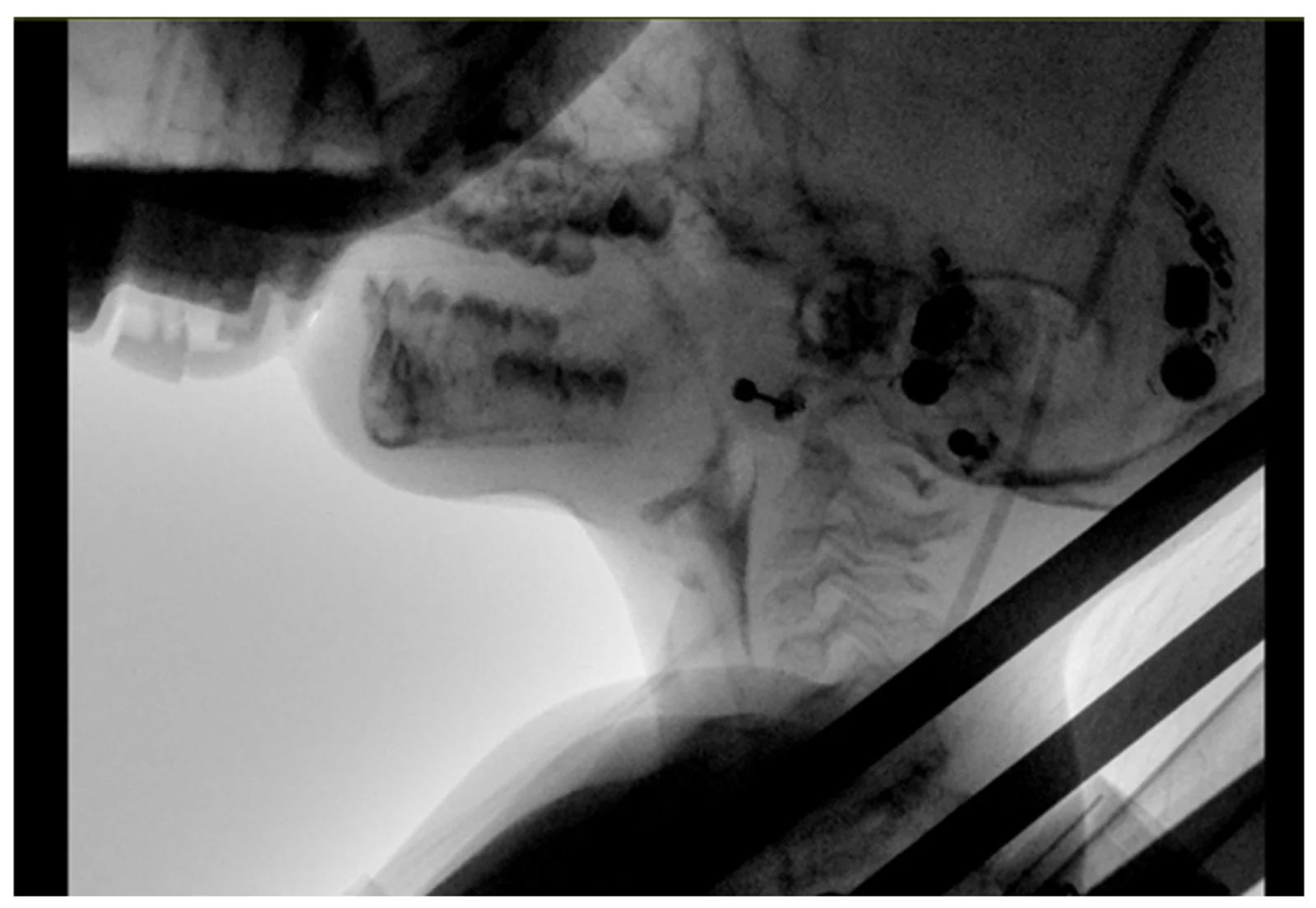
Lateral fluoroscopic image from the MBSS performed at 3 years and 7 months, demonstrating silent aspiration of thin liquid, with contrast material passing below the vocal folds without an observable attempt to eject it.

A follow-up MBSS at 3 years and 7 months demonstrated persistent severe oropharyngeal dysphagia, with aspiration reported across the tested consistencies, including thin liquids, thickened liquids, and purees. A PAS score of 8 was documented, indicating silent aspiration during the first trial (Figure 4). Thin liquids were administered using a milk bottle. Thickened liquid was prepared using one scoop of thickening agent mixed with 300 mL of milk and was also administered using a milk bottle, while 1 mL of puree was administered by syringe. The examination additionally demonstrated premature bolus spillage, delayed swallow initiation, significant vallecular and pyriform sinus residue, and coating of the posterior pharyngeal wall. These findings indicated ongoing persistent swallowing impairment and compromised airway protection, supporting continued reliance on enteral feeding.

At 3 years and 10 months, the patient underwent a fundoplication procedure to address severe GERD that had remained refractory to medical management. G-tube feeding was continued due to swallowing impairment and aspiration risk documented before surgery. No MBSS was performed immediately following fundoplication; the next documented MBSS was conducted at 5 years and 10 months.

Following G-tube placement and fundoplication, a structured oral-feeding and swallowing therapy program was initiated at 4 years of age under the supervision of a senior speech-language pathologist specializing in swallowing disorders. The program initially involved controlled oral trials of 1 mL of thickened liquid four times daily, prepared using one scoop of ThickenUp Clear mixed with 150 mL of water. After one week, the frequency of oral trials could be increased to six times daily, provided that bedside swallowing reassessment was satisfactory and the patient’s medical condition remained stable. Further adjustments to oral intake, including the introduction of small amounts of pureed food, were guided by repeated clinical reassessments and the patient’s overall medical status.

Clinical reassessment, including cervical auscultation, did not identify overt signs suggestive of aspiration during the reported trials. However, given the patient’s documented history of silent aspiration, these findings were interpreted cautiously, as neither cervical auscultation nor the absence of overt clinical signs could exclude aspiration. The protocol allowed oral trials to be modified or paused according to clinical reassessment and medical stability, with ongoing pediatric and pulmonary follow-up. A subsequent MBSS was performed to reassess swallowing function and airway protection and inform further feeding recommendations.

**Figure 5:**
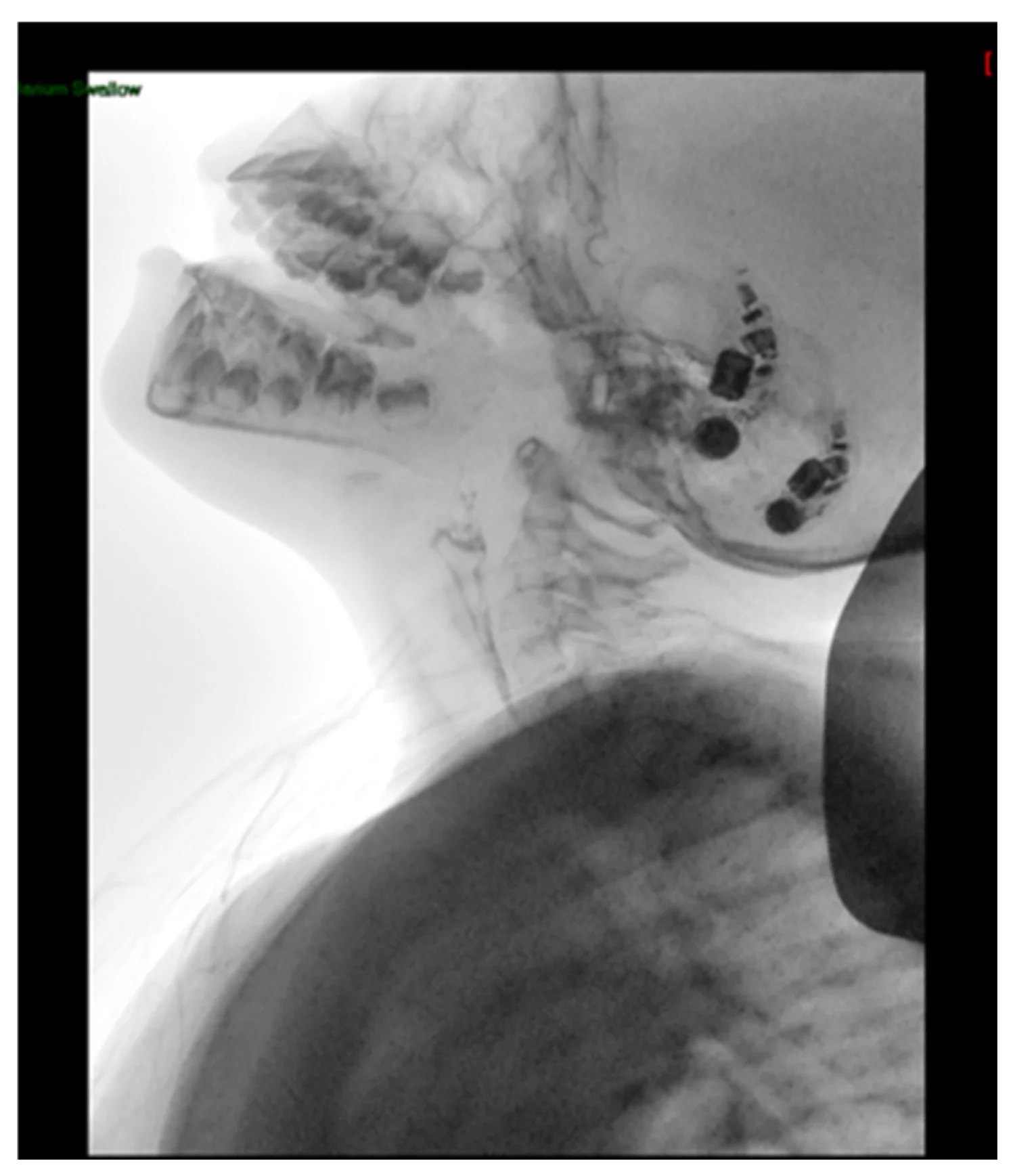
Lateral fluoroscopic image obtained during the MBSS at 5 years and 10 months of age, demonstrating vallecular residue and laryngeal penetration.

At 5 years and 10 months, repeat MBSS demonstrated persistent pharyngeal dysphagia. Thickened liquid was prepared using one scoop of thickening agent mixed with 300 mL of thin liquid and administered by syringe at documented bolus volumes of 1, 3, and 5 mL. Puree was administered using a teaspoon and tablespoon. A single episode of laryngeal penetration was reported during the assessment of thickened liquids and purees, although the specific consistency associated with penetration was not documented. Pharyngeal residue involving the vallecula and pyriform sinuses was also observed (Figure 5). No aspiration was observed with thickened liquids or purees during the assessed trials. However, aspiration persisted with unthickened thin liquids. These findings supported a cautious, consistency-specific approach to oral intake rather than unrestricted feeding.

The most recent MBSS, performed at 6 years of age, demonstrated persistent pharyngeal dysphagia. Thickened liquid was prepared using one scoop of thickening agent mixed with 200 mL of thin liquid and administered by syringe at documented bolus volumes of 1, 3, and 5 mL. Puree was administered using a teaspoon and tablespoon. Unthickened thin liquids were also administered separately by syringe at volumes of 1, 3, and 5 mL, with one trial at each volume. The examination demonstrated anterior and premature bolus spillage, residue involving the soft palate, tongue, vallecula, and pyriform sinuses, coating of the posterior pharyngeal wall, delayed swallow initiation, and reduced laryngeal elevation and hyolaryngeal excursion.

**Figure 6:**
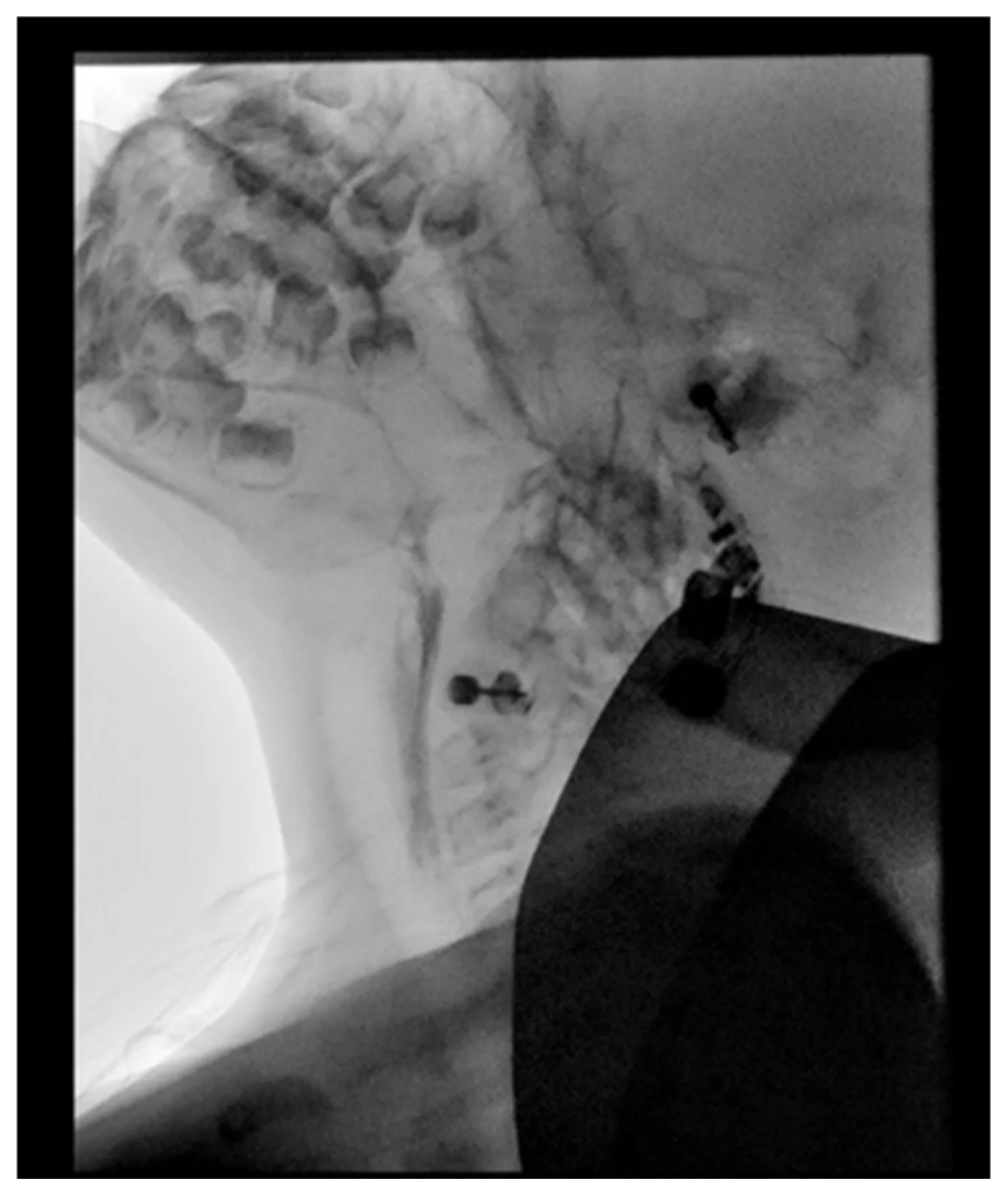
Lateral fluoroscopic image obtained during the MBSS at 6 years of age, demonstrating persistent pharyngeal swallowing abnormalities. No aspiration was observed during the illustrated swallow of thin liquid.

Deep laryngeal penetration (PAS 4) was documented across the tested consistencies (Figure 6); however, no aspiration was observed during the assessed trials. Compared with the findings reported in earlier examinations, the most recent assessment described reduced pharyngeal residue and more timely swallow initiation. Nevertheless, differences in examination conditions and incomplete historical protocol documentation limit direct comparisons across timepoints. Importantly, the absence of observed aspiration during this examination does not establish that aspiration risk had resolved, particularly given the patient’s history of silent aspiration and persistent deep laryngeal penetration. Clinically, the patient tolerated controlled amounts of pureed foods and thickened liquids, while gastrostomy feeding remained necessary for nutritional support. Oral intake continued to be advanced cautiously.

A subsequent gastrointestinal contrast examination performed through the G-tube demonstrated mild gastroesophageal reflux extending to the mid-esophagus in the oblique projection. Gastric filling and emptying were normal, with appropriate gastroesophageal junction anatomy and a normally positioned duodenojejunal flexure. Compared with earlier gastrointestinal imaging demonstrating severe reflux prior to fundoplication, the later examination showed less extensive reflux. However, this difference reflects the reported radiological findings and does not, by itself, establish a causal effect of fundoplication.

### Assessment Methods

Longitudinal dysphagia assessment included serial clinical and instrumental evaluations conducted as part of the patient’s routine clinical care. Bedside swallowing assessments included oral motor and swallowing evaluations using different food textures and liquid consistencies, with cervical auscultation used as an adjunct during selected assessments. Instrumental swallowing assessments were performed using MBSS under videofluoroscopy [13,14]. Available MBSS findings were reviewed longitudinally for documented features of oral and pharyngeal swallowing function, including oral containment and bolus control, swallow initiation, hyolaryngeal excursion, pharyngeal residue, laryngeal penetration, and aspiration. Where documented, airway invasion was characterized using the eight-point Penetration–Aspiration Scale (PAS), which describes the depth of airway invasion and the response to material entering the airway [15,16]. As these assessments were conducted over several years by different treating teams, examination protocols, bolus volumes, administration methods, and the number of trials were not consistently documented across all timepoints. The timeline of key clinical events and interventions is summarized in Table 1. Longitudinal MBSS findings, including tested consistencies, airway invasion, other swallowing findings, and feeding recommendations, are summarized in Table 2.

## 3. Discussion

This case report provides a detailed longitudinal description of swallowing function and dysphagia management in a child with distal trisomy 13q, spanning instrumental evaluations from 18 months to six years of age. Serial assessments documented persistent but changing oral and pharyngeal abnormalities, including silent aspiration during earlier evaluations and the absence of observed aspiration during the final examination under the assessed conditions, despite persistent laryngeal penetration. The case illustrates how repeated instrumental assessment, interpreted alongside clinical findings, informed changes in feeding recommendations over time.

### 3.1. Comparison with Other Chromosomal Disorders

The dysphagia profile observed in this case, characterized by hypotonia, oral and pharyngeal swallowing impairment, and silent aspiration, shares features with those reported in other chromosomal disorders. Although full trisomy 13 (Patau syndrome) is associated with substantial congenital anomalies and high early mortality, clinical outcomes vary according to cytogenetic status, and feeding difficulties have been described among affected children [17,18]. Similarly, feeding and swallowing dysfunction has been reported in children with trisomy 18 (Edwards syndrome) and trisomy 21 (Down syndrome), although the available evidence differs in the extent of instrumental characterization [19,20,21]. The pattern of silent aspiration and pharyngeal swallowing impairment observed in our patient also shares features with findings reported in children with 22q11.2 deletion syndrome [22]. These comparisons suggest that overlapping patterns of swallowing dysfunction may occur across chromosomal disorders despite differences in their genetic etiologies and clinical manifestations.

### 3.2. Potential Contributors to the Dysphagia Phenotype

The dysphagia observed in this patient is likely multifactorial and should be interpreted within the context of her complex neurodevelopmental and medical phenotype. Relevant clinical features included severe hypotonia, global developmental impairment, neurological involvement with seizures, craniofacial abnormalities, and severe GERD. These features are consistent with the range of conditions associated with feeding and swallowing dysfunction in children with genetic and neurodevelopmental disorders [2,3,4,23,24].

The pathogenic 13q22.2–q34 copy-number gain encompasses numerous genes involved in neurodevelopment, including *ZIC2*, which has an established role in central nervous system development [25]. However, a specific genotype–dysphagia relationship has not been established for distal trisomy 13q. Moreover, additional mosaic 13q and chromosome 12q copy-number gains were identified in this patient, and the precise structural mechanism underlying the chromosomal abnormalities remains unresolved. Consequently, the swallowing phenotype cannot be attributed to a specific duplicated gene or chromosomal segment on the basis of this single case. Rather, the genetic findings provide important clinical context for a complex neurodevelopmental phenotype in which dysphagia represents one component of multisystem involvement.

### 3.3. Analysis of Therapeutic Interventions and Serial Assessments

A structured, stepwise therapeutic approach formed an important component of the patient’s longitudinal management. Initiated at four years of age, the intervention involved controlled oral trials of thickened liquids, followed by the gradual introduction of pureed foods, with adjustments guided by clinical reassessment and serial MBSS findings. The individualized approach is consistent with recommendations emphasizing the role of instrumental swallowing assessment in guiding dysphagia management and the careful use of modified liquid consistencies [13,14,26].

Thickened liquids are commonly used in pediatric dysphagia management, although evidence regarding their effects on respiratory outcomes remains limited, emphasizing the importance of individualized assessment and monitoring [27]. Gradual reduction in liquid thickness has been described in selected children with documented aspiration [28]; however, these findings should not be generalized to children with complex neurodevelopmental conditions or interpreted as evidence that a comparable weaning approach was undertaken in the present case.

Serial MBSS examinations documented changes in swallowing findings over the patient’s clinical course. Earlier examinations demonstrated aspiration across the tested consistencies. At 5 years and 10 months, aspiration persisted with unthickened thin liquids, whereas no aspiration was observed with thickened liquids or purees during the assessed trials. At six years of age, no aspiration was observed during the assessed trials, including those involving unthickened thin liquids, although deep laryngeal penetration and other pharyngeal swallowing abnormalities persisted.

These findings suggest changes in airway protection under the specific conditions assessed at different timepoints. However, differences in tested consistencies, bolus volumes, examination protocols, and the completeness of historical documentation limit direct comparisons across examinations. Consequently, the findings should not be interpreted as demonstrating a quantified improvement in swallowing physiology or resolution of aspiration risk.

The absence of observed aspiration during the earlier gastrointestinal contrast examination at 13 months should not be interpreted as evidence of safe oropharyngeal swallowing. Gastrointestinal contrast studies and MBSS have different assessment objectives, and the absence of aspiration during gastrointestinal imaging does not reliably exclude aspiration identified during dedicated swallowing assessment [29].

The observed changes occurred alongside swallowing intervention, neurodevelopmental maturation, medical management, and surgical treatment of severe GERD. Following Nissen fundoplication at 3 years and 10 months of age, subsequent gastrointestinal imaging documented less extensive reflux than earlier examinations. However, no MBSS was performed between fundoplication and the subsequent swallowing assessment at 5 years and 10 months, and the available findings do not establish that fundoplication directly improved swallowing function or airway protection. The relative contributions of these concurrent factors cannot be determined from this single case.

The principal clinical contribution of the longitudinal assessments was their role in informing individualized feeding recommendations and monitoring swallowing function over time, while recognizing the continued need for cautious oral intake and enteral nutritional support.

### 3.4. Clinical Implications and Future Directions

This case illustrates the potential value of repeat instrumental swallowing assessment when clinically meaningful changes occur or when findings are expected to influence management decisions. In this patient, serial MBSS provided objective information regarding airway protection, pharyngeal residue, swallow initiation, and swallowing performance across the tested consistencies, which informed decisions regarding continued enteral feeding and cautious progression of oral intake. The timing of reassessment should be individualized according to clinical status, developmental changes, and the likelihood that results will alter management, while minimizing unnecessary radiation exposure [13,14,30].

Regarding long-term prognosis, full oral feeding may not be a realistic goal for all children with severe swallowing impairment. Management should therefore balance adequate nutrition and airway protection, including continued G-tube feeding when indicated, with opportunities for positive oral experiences using consistencies selected according to instrumental findings and clinical reassessment [3]. Future multicenter studies and systematic collection of longitudinal swallowing data in rare chromosomal disorders may help identify prognostic indicators and inform individualized management strategies.

### 3.5. Limitations

This report has several limitations. First, as a single-case report, the observed clinical trajectory cannot be generalized to other children with distal trisomy 13q, whose phenotypes may vary substantially according to the extent and nature of the chromosomal abnormality. Second, the retrospective longitudinal nature of the case resulted in variability in the timing and available documentation of swallowing assessments. As evaluations were conducted as part of routine clinical care by different treating teams, tested consistencies, bolus volumes, administration methods, trial counts, and standardized quantitative swallowing measures were not consistently documented or comparable across all historical timepoints. The availability and application of quantitative videofluoroscopic swallowing measures in pediatric populations have also been discussed in the literature [31]. The intervals between examinations further limited the ability to characterize changes in swallowing function over time, although the need to minimize unnecessary radiation exposure remains an important consideration when planning repeated MBSS examinations in pediatric patients.

An additional limitation concerns the cytogenetic characterization of the patient’s chromosomal abnormality. Although genomic testing identified a pathogenic terminal 13q22.2–q34 copy-number gain consistent with distal trisomy 13q and conventional chromosome analysis demonstrated 47, XX, add (12) (q24.31), +mar, the precise structural mechanism underlying the abnormalities could not be established from the available investigations. Further cytogenetic characterization and parental chromosome studies were recommended but were not available. Consequently, it cannot be determined whether the rearrangement occurred de novo or was inherited from a parental balanced rearrangement, and no conclusions regarding recurrence risk can be drawn. The genetic findings are therefore presented as clinical context for the patient’s dysphagia phenotype rather than as a comprehensive cytogenetic characterization.

Finally, changes in documented swallowing findings occurred alongside multiple interventions and ongoing development; therefore, causal effects cannot be attributed to any individual therapeutic, medical, or surgical intervention. Despite these limitations, the longitudinal clinical and instrumental observations provide a detailed account of dysphagia management in a rarely described neurogenetic condition.

## 4. Conclusions

This case illustrates the complexity of longitudinal dysphagia management in a child with distal trisomy 13q. Serial clinical and instrumental swallowing assessments documented changes in swallowing findings over time and supported individualized decision-making regarding enteral nutrition, texture modification, therapeutic intervention, and cautious progression of oral intake. Although no aspiration was observed during the final MBSS under the conditions assessed, persistent deep laryngeal penetration and the history of silent aspiration indicated that swallowing safety remained a concern. Differences in assessment conditions and concurrent clinical interventions preclude definitive conclusions regarding the extent or causes of these changes. This case highlights the value of multidisciplinary management and clinically indicated reassessment in children with complex neurodevelopmental disorders. Further reports are needed to characterize dysphagia phenotypes and longitudinal outcomes in distal trisomy 13q.

## Figures and Tables

**Table 1:** Timeline of key clinical events and interventions.

Age/Timepoint	Key Clinical Events
Birth-3 months	Global developmental delay noted in early infancy; onset of seizures at 3 months.
13 months	Initial referral to the swallowing team; clinical swallowing assessment and Barium Swallow Study performed.
18 months	First MBSS: aspiration across tested consistencies; G-tube feeding recommended.
22 months	Barium Meal Study performed to evaluate severe GERD.
3 years 7 months	MBSS: persistent aspiration across tested consistencies; PAS score of 8 documented.
3 years 10 months	Fundoplication performed for refractory GERD; G-tube feeding continued.
4 years	Structured oral-feeding and swallowing therapy initiated, beginning with controlled trials of thickened liquid; purees introduced subsequently according to clinical reassessment and medical stability.
5 years 10 months	MBSS: laryngeal penetration observed; no aspiration with thickened liquids or purees during assessed trials; but aspiration persisted with unthickened thin liquids.
6 years	MBSS: deep laryngeal penetration persisted (PAS 4); no aspiration observed during the assessed trials, including unthickened thin-liquid trials.

Abbreviations: MBSS, modified barium swallow study; PAS, Penetration-Aspiration Scale; GERD, gastroesophageal reflux disease; G-tube, gastrostomy tube.

**Table 2:** Longitudinal MBSS findings.

Age/Timepoint	Consistencies and Administration	Airway Invasion	Other Swallowing Findings	Feeding Recommendations
18 months	Thin liquid by bottle; thickened liquid (1 scoop of thickener in 300 mL milk) by bottle; puree (1 mL) by syringe. Number of trials: Not documented.	Aspiration across tested consistencies; PAS 8 documented. Consistency-specific PAS scores: Not documented.	Premature bolus spillage, delayed swallow initiation, significant vallecular and pyriform sinus residue, and posterior pharyngeal wall coating. Residue clearance: Not documented.	G-tube feeding recommended.
3 years 7 months	Thin liquid by bottle; thickened liquid (1 scoop of thickener in 300 mL milk) by bottle; puree (1 mL) by syringe. Number of trials: Not documented.	Aspiration across tested consistencies; silent aspiration (PAS 8) documented during the first trial. Consistency-specific PAS scores: Not documented.	Premature bolus spillage, delayed swallow initiation, significant vallecular and pyriform sinus residue, and posterior pharyngeal wall coating. Residue clearance: Not documented.	Continued enteral feeding recommended.
5 years 10 months	Thickened liquid (1 scoop of thickener in 300 mL thin liquid) administered by syringe at 1, 3, and 5 mL; puree by teaspoon and tablespoon. Unthickened thin liquid also assessed; administration method, bolus volumes, and number of trials: Not documented.	One episode of laryngeal penetration during the assessment of thickened liquids and purees. No aspiration observed with thickened liquids or purees; aspiration observed with unthickened thin liquids. Consistency responsible for penetration: Not documented.	Vallecular and pyriform sinus residue. Residue clearance: Not documented.	Controlled oral intake of thickened liquids and purees alongside G-tube feeding.
6 years	Thickened liquid (1 scoop of thickener in 200 mL thin liquid) administered by syringe at 1, 3, and 5 mL; puree by teaspoon and tablespoon. Unthickened thin liquid administered separately by syringe at 1, 3, and 5 mL, with one trial per volume. Trial counts for thickened liquid and puree: Not documented.	Deep laryngeal penetration across tested consistencies (PAS 4); no aspiration observed during the assessed trials.	Anterior and premature bolus spillage, delayed swallow initiation, reduced laryngeal elevation and hyolaryngeal excursion, residue involving the soft palate, tongue, vallecula, and pyriform sinuses, and posterior pharyngeal wall coating. Residue clearance: Not documented.	Continued cautious oral intake of controlled amounts of thickened liquids and purees alongside G-tube nutritional support.

Abbreviations: MBSS, modified barium swallow study; PAS, Penetration–Aspiration Scale; G-tube, gastrostomy tube.

## Data Availability

The data supporting the findings of this case report are contained within the article. Additional patient-level clinical data are not publicly available due to privacy and confidentiality considerations.

## Abbreviations

The following abbreviations are used in this manuscript:

CMA	Chromosomal microarray analysis
DJ	Duodenojejunal
GERD	Gastroesophageal reflux disease
G-tube	Gastrostomy tube
MBSS	Modified Barium Swallow Study
NGT	Nasogastric tube
PDA	Patent ductus arteriosus
